# Problematic internet use among adolescents in Sousse, Tunisia: Prevalence and correlates

**DOI:** 10.1192/j.eurpsy.2025.928

**Published:** 2025-08-26

**Authors:** R. Ayoub, M. Fakhfekh, M. Ajmi, A. Lajnef, M. Oueslati, A. Guedria

**Affiliations:** 1 Child and Adolescent Psychiatry Department, Fattouma Bourguiba university Hospital; 2 University of Monastir, Monastir, Tunisia

## Abstract

**Introduction:**

Cyberaddiction, characterized by excessive and uncontrollable internet use, has been linked to various psychological and behavioral issues. Adolescents are particularly vulnerable to these risks, as they are heavy users of digital platforms, often spending long hours online for social interaction, entertainment, and gaming. While research on this issue is expanding, our study seeks to contribute further insights into the factors associated with problematic internet use and its implications for adolescent well-being.

**Objectives:**

The objective of our study was to determine the prevalence of cyberaddiction among adolescents in Sousse, Tunisia and to investigate its relationship to self-esteem.

**Methods:**

We conducted a descriptive cross-sectional study with analytical objectives among adolescents aged 12 to 18 from schools in Sousse Medina, Tunisia during February, March, and April 2024. We used a demographic information form, the Young Internet Addiction Test and the Rosenberg Self-Esteem Scale for data collection.

**Results:**

Our population consisted of 416 adolescents. The most represented age group was 14 years old (22.1%), with a mean age of 15.05 ± 1.7 years. The sample comprised 236 females (56.7%) and 180 males (43.3%). Our findings indicate that 83.2% of participants exhibited problematic internet use with potential life consequences, 11.3% showed occasional excessive use but maintained control, and 5.5% experienced severe repercussions from their internet use. Additionally, 43% of participants spent more than 2 hours per day online. A statistically significant relationship was observed between the amount of time spent online and cyberaddiction (p = 0.009, with higher levels of cyberaddiction associated with increased internet usage. According to the Rosenberg Scale, 51.9% of adolescents had low self-esteem, however, no statistically significant relationship was observed between self-esteem and cyberaddiction.

**Conclusions:**

Our findings highlight a concerning prevalence of problematic internet use among adolescents, with no significant correlation identified between self-esteem and cyberaddiction. This suggests that other underlying factors may contribute to the challenges faced by this population. It is crucial for healthcare professionals and policymakers to prioritize preventive measures and awareness campaigns, promoting healthier online habits and ultimately fostering the well-being of young people in an increasingly digital world.

**Disclosure of Interest:**

None Declared

